# Adjuvant roles of interleukin-7 in enhancing T cell recovery during antiretroviral therapy for individuals with HIV: a systematic review and meta-analysis

**DOI:** 10.1080/07853890.2025.2594303

**Published:** 2025-12-01

**Authors:** Bixin Deng, Xia Qiu, Ruixi Zhou, Tiechao Ruan, Wenting Lu, Dezhi Mu

**Affiliations:** ^a^ Department of Pediatrics, West China Second University Hospital, Sichuan University, Chengdu, China; ^b^ Key Laboratory of Birth Defects and Related Diseases of Women and Children, Sichuan University, Ministry of Education, Chengdu, China; ^c^ Integrated Care Management Center, West China Hospital, Sichuan University, Chengdu, China

**Keywords:** IL-7, HIV, CD4^+^ T cell counts, CD8^+^ T cell counts, HIV-DNA load

## Abstract

**Background:**

T cell suppression has been observed in individuals with human immunodeficiency virus (HIV) undergoing antiretroviral therapy (ART). This study aimed to evaluate the effect of interleukin (IL)-7 on T cell count and HIV DNA load in individuals with HIV receiving long-term ART.

**Methods:**

We screened English-language articles on PubMed, Embase, Cochrane, and Web of Science from inception up to April 7, 2023. The included articles were assessed using the Cochrane risk-of-bias assessment tool. A meta-analysis of CD4^+^ and CD8^+^ T cell counts and HIV DNA load was performed in individuals with HIV before and after IL-7 administration.

**Results:**

Eight articles were included in our study. CD4^+^ and CD8^+^ T cell counts were significantly elevated at weeks 4 and 12 after administration of 20 μg/Kg IL-7 in individuals with HIV on long-term ART. The HIV DNA load in whole blood also increased after IL-7 treatment; however, no significant change was observed in intracellular DNA in peripheral blood mononuclear cells and CD4^+^ T cells. Moreover, IL-7 is generally well tolerated in clinical studies.

**Conclusion:**

Our analysis revealed that IL-7 can induce an increase in CD4^+^ and CD8^+^ T cell counts in individuals with HIV receiving long-term ART, aiding in immune system reconstitution and increasing the HIV DNA load in the whole blood. These findings suggest that while IL-7 holds promise as an adjuvant strategy for immune restoration, its long-term impact on HIV persistence must be weighed in clinical application.

## 1. Introduction

As a global pandemic, the high pathogenicity and mortality rates of Human immunodeficiency virus (HIV) pose significant global healthcare problems that must be addressed urgently [1]. Pathogenically, HIV infection is characterized by the progressive depletion of CD4^+^ T cells and a decline in immune surveillance, which results in severe opportunistic infections and malignancies [2]. Aside from reduction, as the primary targets of HIV infection [3], CD4^+^ T cells also exhibit reduced proliferative capacity and helper functions as well as immune senescence [4]. Currently, antiretroviral therapy (ART) effectively reduces the HIV viral load and delays its progression to acquired immune deficiency syndrome (AIDS), making it the main treatment for HIV infections [5]. However, individuals with HIV infection require lifelong treatment owing to the persistence of a stable latent viral reservoir that remains unaffected by ART [6]. Moreover, several reports have shown that CD4^+^ T cells do not recover in a small proportion of individuals living with HIV after long-term ART despite significant viral load control [7]. Therefore, it is necessary to identify an adjuvant that can help restore CD4^+^ T cells and rebuild the immune system in individuals with HIV during long-term ART.

Once ART is interrupted, integrated HIV within cells such as CD4^+^ T cells continues to replicate, which is the fundamental reason that ART cannot cure HIV [8]. Interleukins (IL) play important roles in the early development, homeostasis, and proliferation of T cells [9]. Therefore, IL-based immunotherapy has been proposed for the treatment of HIV to increase T cell counts and further assist in immune reestablishment and virus eradication [10]. IL-2, when used in combination with ART, has been found to significantly impact the recovery of CD4^+^ T cell counts [11,12]. However, IL-2 as an adjuvant has not only failed to reduce the mortality rate among individuals with HIV but has also been associated with an increase in grade 4 clinical events [13].

IL-7, a 25-kDa soluble globular protein, was firstly discovered in the last century [14], with a structure of four alpha helices and a hydrophobic core [15]. It is well known that IL-7 is essential for maintaining immune system homeostasis, and specifically, in the development, proliferation, and differentiation of B and T cells [16]. It has also been reported that IL-7 therapy can enhance immune reconstitution and increase CD4 levels in both acute and chronic infectious diseases, including septic shock and COVID-19 [17]. Additionally, IL-7 is notably inversely correlated with CD4^+^ T cell count in HIV disease [18]. Consequently, IL-7 is considered a potential immune-based therapeutic for HIV disease [19], due to its high efficacy in stimulating the transcription and replication of HIV integrated into CD4^+^ T cells and peripheral blood mononuclear cells (PBMCs), targeting widespread HIV-1 strains [8]. In addition, a temporary increase in HIV replication was observed during IL-7-assisted ART, which returned to baseline post-trial. There is a lack of systematic review and meta-analyses on the efficacy of IL-7 in antiretroviral therapy for individuals with HIV. Therefore, we conducted a systematic review and meta-analysis to assess the clinical efficacy and safety of IL-7 as an immunotherapy agent for HIV infection.

## 2. Methods

### 2.1. Search strategy and selection criteria

The study protocol followed the Preferred Reporting Items for Systematic Reviews and Meta-Analyses-Diagnostic Test Accuracy Studies (PRISMA-DTA) statement (Supplemental Table S1) [20]. The process also followed the requirements of the PRISMA 2020 statement [21]. The study was registered in the PROSPERO database (ID: CRD42023416894) in April 2023. We searched the database using the following terms: ‘HIV’ or ‘human immunodeficiency virus,’ cross-referenced with ‘IL-7’ or ‘Interleukin-7.’ The PubMed, Embase, Cochrane, and Web of Science databases were searched for potentially relevant English language studies (from inception up to April 7, 2023). The complete search strategy was provided in the Supplementary Materials. The references of the identified articles were also reviewed to supplement the data sources.

Study eligibility for inclusion consisted of the following criteria: (1) the study design was either a randomized trial, quasi-experimental or cohort/case–control study, (2) study population consisted of individuals with HIV administrating ART, (3) the intervention group was treated with IL-7 as adjuvant therapy, (4) the comparator group did not receive any adjuvant therapy, (5) reported outcomes included greater than or equal to 1 of the following: CD4^+^ T cell counts, CD8^+^ T cell counts, and HIV-DNA load. Exclusion criteria included: (1) studies only available as an abstract and (2) full-text articles published in non-English languages.

### 2.2. Study selection

The titles and abstracts were independently screened by two reviewers (B.D. and X.Q.) after removing duplicate studies retrieved from the four databases and other sources [22]. Subsequently, the full texts were reviewed based on the inclusion criteria. Disagreements were resolved through consultation and third-party opinions were sought if necessary.

Full-text studies with participants diagnosed with HIV who received ART for at least 6 months and received exogenous IL-7 as an adjunctive therapy to ART were included to further extract data for meta-analysis. Reviews, abstracts, comments, case reports, non-English-language publications, and studies with fewer than five participants were excluded.

### 2.3. Risk of bias assessment

Two reviewers (B.D. and X.Q.) independently assessed the risk of bias in eligible studies using the Cochrane risk-of-bias assessment tool [11]. which assesses each study in the following domains: selection, performance, detection, attrition, reporting, and other biases. Reviewers rated each domain as low, unclear, or high risk, and ReviewManager (RevMan) was used to generate a ‘risk of bias graph’ and ‘risk of bias summary.’ The two reviewers discussed and resolved any disagreements, consulting third-party (R.Z.) opinions if necessary.

### 2.4. Data extraction and statistical analysis

Two reviewers (B.D. and X.Q.) independently extracted data from the eligible studies to establish standardized collection forms, including the following: author, year of publication, trial ID, participants (age, HIV-RNA levels, duration under treatment with ART, baseline CD4^+^ T cells, and type of virus), IL-7 administration (route and dose), CD4^+^ and CD8^+^ T cell counts, HIV DNA load in whole blood, PBMCs, and CD4^+^ T cells, and adverse events (AEs). The mean and standard deviation (SD) were calculated for CD4^+^ and CD8^+^ T cell counts, and HIV DNA load at week 4/12 after IL-7 administration and before administration. Disagreements were resolved through consultation and third-party (R.Z.) opinions were sought if necessary.

Data analysis was performed using RevMan software. Meta-analysis was performed on the following data from individuals with HIV undergoing ART before and after IL-7 immunotherapy: 1) CD4^+^ T cell count in the peripheral blood, 2) CD8^+^ T cell count in the peripheral blood, and 3) HIV DNA load in whole blood, PBMCs, and CD4^+^ T cells. Subgroup analysis was conducted according to IL-7 dose (3, 10, 20, and 30 μg/Kg) and detection time (weeks 4 and 12). An I-squared (I^2^) > 50% was considered inappropriate heterogeneity, in which case the random-effects model was used [23]. Statistical significance was set at *p* < 0.05.

## 3. Results

### 3.1. Search results

**Figure 1.**
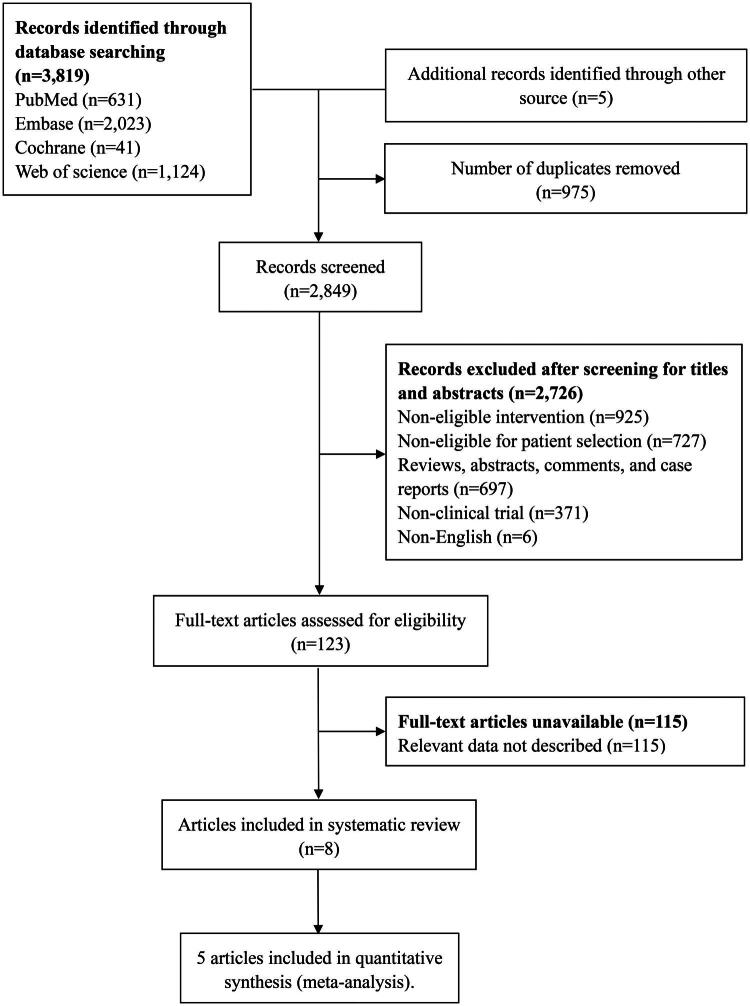
Flow diagram of study selection.

A total of 3,819 records were retrieved from the four databases based on the search terms, and 5 records were retrieved from other sources (the relevant references from published meta-analyses). After removing 975 duplicate records, the titles and abstracts of the remaining 2,849 records were screened. We excluded 2,726 records for the following reasons: ineligible interventions (IL-2, HIV vaccine, etc.), ineligible individual selection (tuberculosis, monkeypox, etc.), reviews, abstracts, non-clinical trials, and non-English articles. The full texts of the remaining 123 records were reviewed and 115 were excluded because relevant data were not described. A total of 8 articles were included in the systematic review [24–31], and 5 of them extracted available data for meta-analysis [24,26–28,31] (Figure 1).

### 3.2. Study characterisations

Table 1 lists the main characteristics of the eight included articles. All were published between 2009 and 2018, all were clinical trials (IDs are listed), and all participants were adults aged 27–66 years old. The participants were infected with the HIV-1 strain (one article did not mention the strain) and had HIV-RNA levels < 50 copies/mL (one article reported < 40 copies/mL) after long-term ART. Participants had baseline CD4^+^ T cell counts ranging from 101 to 705 cells/μL. In all included articles the type of IL-7 used was rhIL-7, which was administered subcutaneously at doses ranging from 3 to 30 μg/Kg (mainly 20 μg/Kg), and the time point of administration was within 2 weeks (mainly on days 0, 7, and 14).

**Table 1.** Main characteristics of included studies.

Author	Year of publication	Trial ID	Participants					IL-7 administration			
			Age	HIV-RNA (copies/mL)	Time under treatment with ART	Baseline CD4^+^ T cells/μL	Type of virus	Route	Type	Dose	Measurements/endpoints
Logerot	2018	CLI-107-14-III	37–52	<50	At least 24 months	213–320	HIV-1	SC	rhIL-7	20µg/Kg on days 0, 7, 14	HIV-DNA load
Wang	2018	CLI-107-14-II	30–55	<40	At least 6 months	101–400	NA	SC	rhIL-7	20µg/Kg on days 0, 7, 14	CD8 T cells
Katlama	2016	NCT01019551-II (Registered on 25 Nov. 2009)	42–56	<50	At least 36 months	462–701	HIV-1	SC	rhIL-7	20µg/Kg on days 0, 7, 14	HIV DNA load
Thiébaut	2016	CLI-107-14-II NCT0119011	42–51	<50	At least 12 months	191–320	HIV-1	SC	rhIL-7	20µg/Kg on days 0, 7, 14	CD4 counts, safety
Sereti	2014	NCT01190111-II	41–51	<50	4–17 years	191–329	HIV-1	SC	rhIL-7	20µg/Kg on days 0, 7, 14	Not mentioned
Lévy	2012	2006–00624-20A/NCT0047732	27–66	<50	At least 12 months	203–344	HIV-1	SC	rhIL-7	10/20/30 µg/Kg on days 0, 7, 14	Safety
Lévy	2009	2004-003772-11A	29–65	<50	2–16 years	107–312	HIV-1	SC	rhIL-7	3/10 µg/Kg on days 0, 2, 4, 6, 8, 10, 12, and 14	Safety
Sereti	2009	NCT00099671	41–48	<50	At least 12 months	323–705	HIV-1	SC	rhIL-7	3, 10, 30, 60, and 100 g/kg on day 0	Not mentioned

ART, antiretroviral therapy; HIV, human immunodeficiency virus; IL, interleukin; NA, not available; rhIL-7: recombinant human IL-7; SC, subcutaneous.

### 3.3. Risk of bias in studies

We assessed the risk of bias for the included articles, and the results are summarized in Supplementary Figure S1. ‘Random sequence generation,’ ‘blinding of participants and personnel,’ ‘blinding of outcome assessment,’ and ‘selective reporting’ in all eight articles [24–31] were assessed as low-risk. Regarding ‘allocation concealment,’ the risk of bias in five articles [24,25,27,29,30] was assessed as unclear. For ‘incomplete outcome data,’ bias in two articles [29,30] was assessed as high risk. Concerning ‘other bias,’ one article [24] was considered as high risk, while the three articles [27,29,30] were assessed as unclear risk. In general, the risk of bias was low.

### 3.4. Increased CD4^+^ T cell counts after IL-7

**Figure 2.**
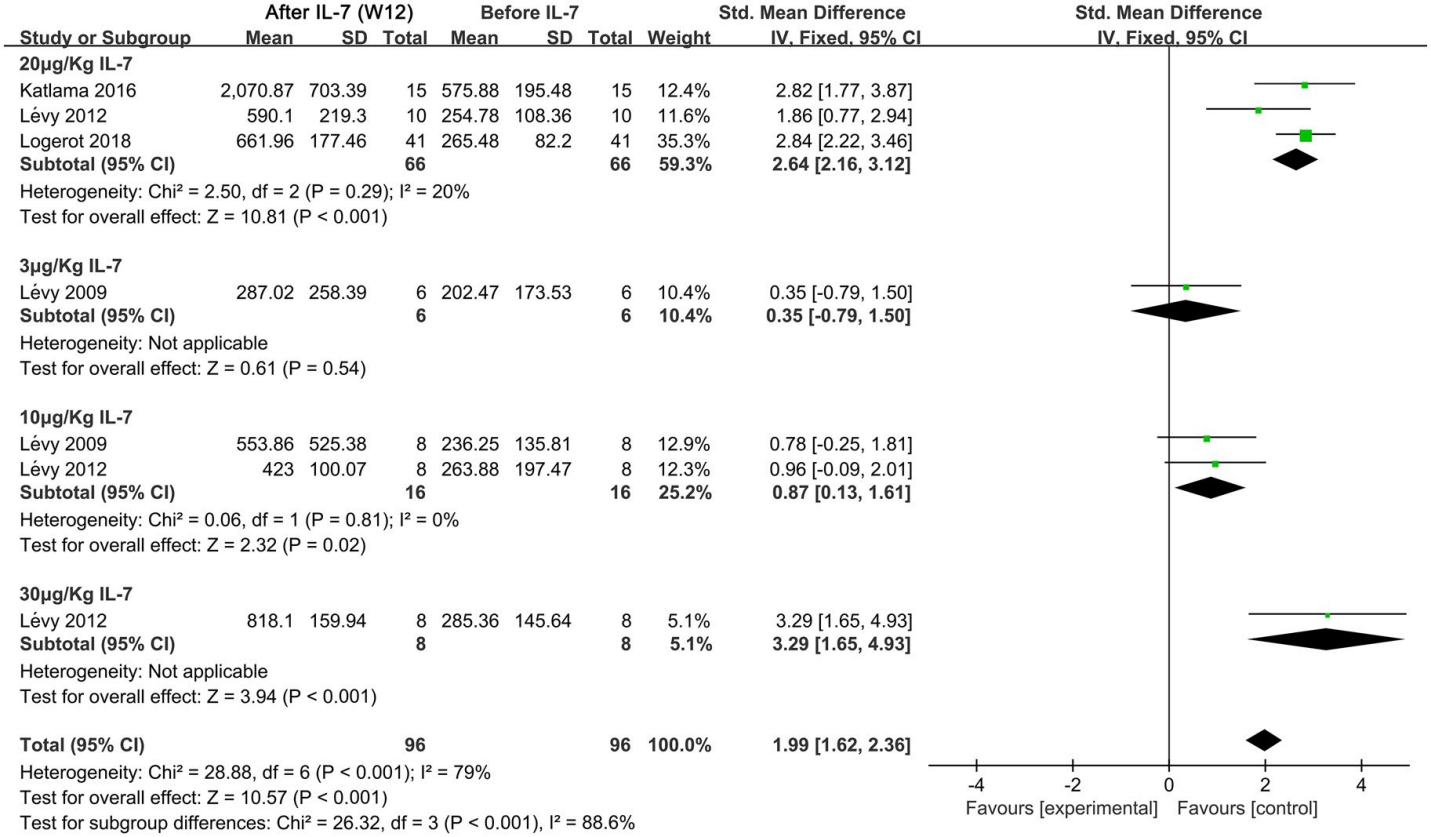
Meta-analysis of CD4^+^ T cell counts at week 12 after IL-7 administration and before administration in individuals with HIV receiving long-term ART. ART, antiretroviral therapy; Chi^2^, Chi-square test; CI, confidence interval; df, degrees of freedom; IL-7, Interleukin-7; IV, inverse variance; I^2^, I-squared; SD, standard deviation; Std., standard deviation; W 12, week 12.

We analyzed CD4^+^ T cell counts in the peripheral blood of individuals with HIV before and after the administration of IL-7 [24, 27, 32, 33] (Figure 2). In general, at week 12 following IL-7 administration, CD4^+^ T cell counts were significantly higher than that before IL-7 administration (standardized mean difference (SMD), 1.99; 95% confidence interval (CI), 1.65–4.93; *p* < 0.001; I^2^ = 79%). Specifically, data from the four included studies showed that, prior to the intervention, the average CD4^+^ T cell count was approximately 290 cells/µL, which increased by 2 to 3 times after the intervention, reaching an average of around 770 cells/µL [24,26–28]. Owing to the high heterogeneity (I^2^ = 88.6%), we performed a subgroup analysis of IL-7 doses. The results suggested that 20 μg/Kg IL-7 significantly increased CD4^+^ T cell counts (SMD, 2.64; 95% CI, 2.16–3.12; *p* < 0.001; I^2^ = 20%) (Figure 2). In addition, at week 4 after IL-7 administration [24,26–28], subgroup analysis revealed a significant difference in CD4^+^ T cell counts only at the 20 μg/Kg dose (SMD, 2.89; 95% CI, 2.33–3.46; *p* < 0.001), 10 μg/Kg (SMD, 1.29; 95% CI, 0.50–2.08; *p* = 0.001), and 30 μg/Kg (SMD, 3.18; 95% CI, 1.58–4.78; *p* < 0.001), with no significant difference at 3 μg/Kg [24,26–28] (Supplementary Figure S2). Additionally, a dose-response effect was observed among the three doses used in this meta-analysis. The 3 μg/Kg dose did not lead to an increase in CD4^+^ T cell counts at either week 4 or 12, which may have contributed to the significant rise in heterogeneity when the results were pooled. These findings suggest that 20 and 30 μg/Kg IL-7, as an adjuvant to ART for HIV, can effectively increase CD4^+^ T cell counts at both weeks 4 and 12.

### 3.5. Increased CD8^+^ T cell counts after IL-7

**Figure 3.**
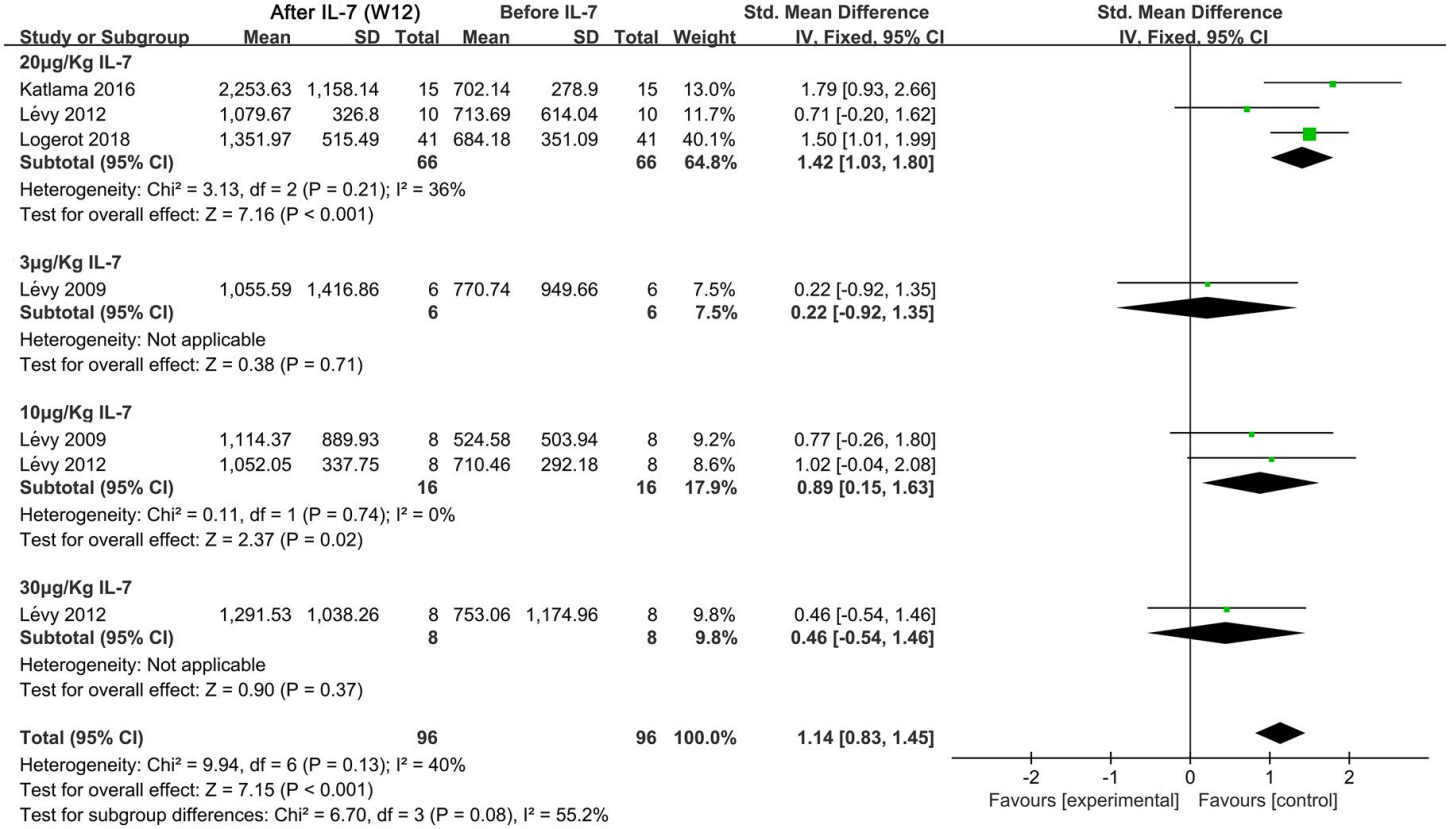
Meta-analysis of CD8^+^ T cell counts at week 12 after IL-7 administration and before administration in individuals with HIV receiving long-term ART. ART, antiretroviral therapy; Chi^2^, Chi-square test; CI, confidence interval; df, degrees of freedom; IL-7, Interleukin-7; IV, inverse variance; I^2^, I-squared; SD, standard deviation; Std., standard deviation; W 12, week 12.

CD8^+^ T cells are important components of the immune system, contributing to the suppression of the virus and the restoration of CD4^+^ T cell counts in individuals with HIV undergoing long-term ART [32]. We observed a significant increase in CD8^+^ T cell counts at week 12 following IL-7 treatment compared to before (SMD, 1.14; 95% CI, 0.83–1.45; *p* < 0.001; I^2^ = 40%). Subgroup analysis suggested that only 20 μg/Kg (SMD, 1.42; 95% CI, 1.03–1.80; *p* < 0.001) and 10 μg/Kg (SMD, 0.89; 95% CI, 0.15–1.63; *p* < 0.02) IL-7 increased CD8^+^ T cell counts, while doses of 3 and 30 μg/Kg did not have a significant effect [24,26–28] (Figure 3). At week 4 after IL-7 administration, only 20 μg/Kg (SMD, 1.79; 95% CI, 1.32–2.25; *p* < 0.001) IL-7 increased CD8^+^ T cell counts, while doses of 3, 10, and 30 μg/Kg did not have a significant effect [24,27,28] (Supplementary Figure S3). These findings suggest that as an adjuvant to ART for HIV, 20 μg/Kg IL-7 can effectively increase CD8^+^ T cell counts at both weeks 4 and 12.

### 3.6. Increased HIV-DNA load in the whole blood after IL-7

**Figure 4.**
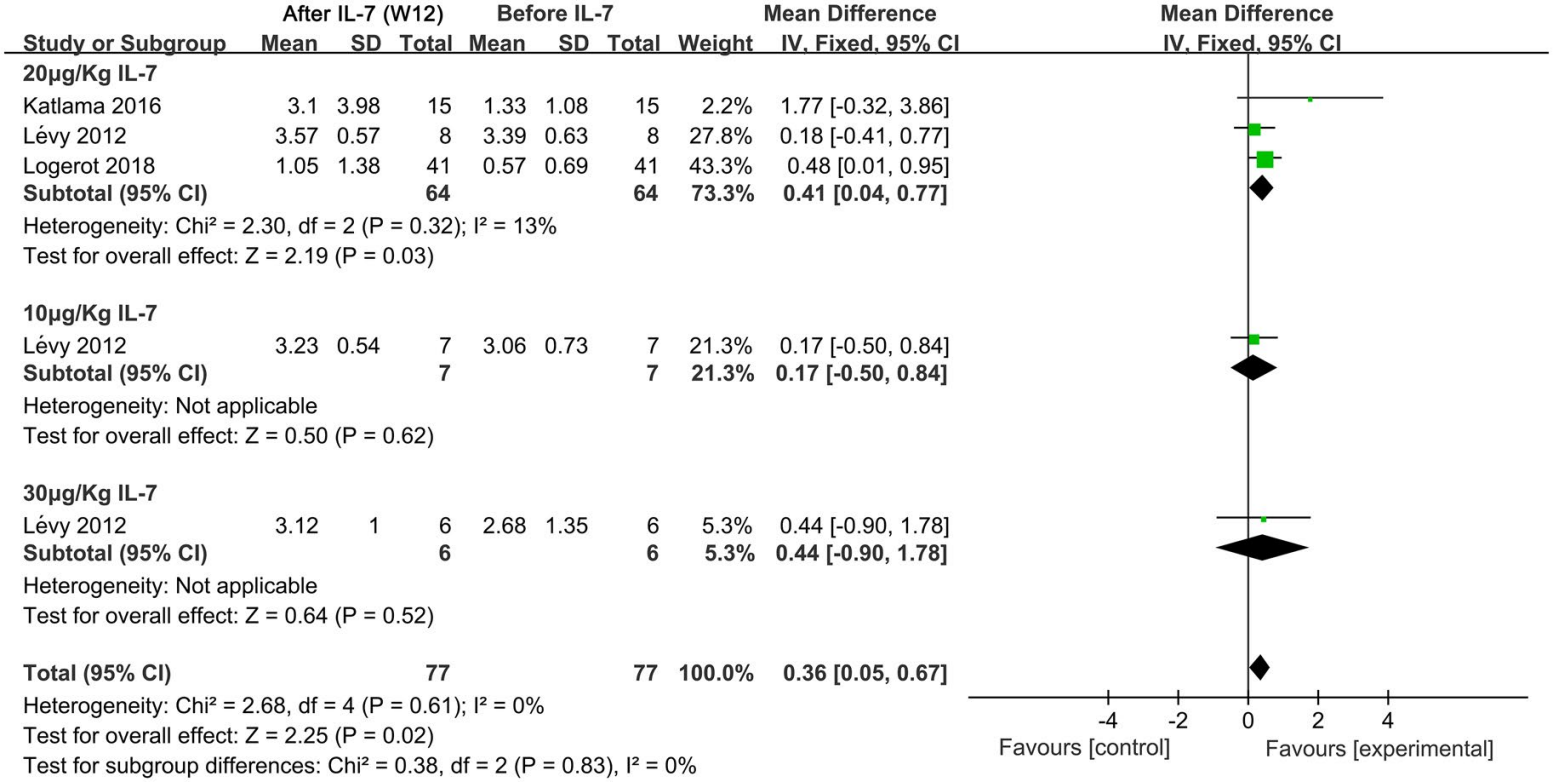
Meta-analysis of HIV DNA load in whole blood at week 12 after IL-7 administration and before administration in individuals with HIV receiving long-term ART. Chi^2^, Chi-square test; CI, confidence interval; df, degrees of freedom; IL-7, Interleukin-7; IV, inverse variance; I^2^, I-squared; SD, standard deviation; W 12, week 12.

HIV DNA load is a fundamental measure of treatment effectiveness [33]. Therefore, we performed a meta-analysis of the HIV DNA loads in whole blood (copies/μl), CD4^+^ T cells (copies/10^6^ CD4^+^ T cells), and PBMCs (copies/10^6^ PBMCs) from individuals with HIV. Notably, at week 12 following IL-7 administration, the HIV DNA load in the whole blood was elevated (SMD, 0.36; 95% CI, 0.05–0.67; *p* = 0.02; I^2^ = 0%). This increase was observed specifically in the 20 μg/Kg subgroup (SMD, 0.41; 95% CI, 0.04–0.77; *p* = 0.03; I^2^ = 13%), with no significant change noted at doses of 10 and 30 μg/Kg [24,26,28] (Figure 4). Similar findings were observed at week 4 after IL-7 administration, where 20 μg/Kg IL-7 resulted in an increase in HIV DNA load in whole blood (SMD, 2.23; 95% CI, 1.27–3.19; *p* < 0.001; I^2^ = 0%) [24,28] (Supplementary Figure S4). In addition, IL-7 did not affect the HIV DNA load in CD4^+^ T cells or PBMCs at any time or dose (Supplementary Figures S5–S8). These results suggest that only 20 μg/Kg IL-7 could cause an increase in whole blood HIV DNA load at weeks 4 and 12, while other doses or time points did not lead to changes in the HIV DNA load in CD4^+^ T cells and PBMCs. Notably, given the increase in total HIV DNA loads and CD4^+^ T cells, the relative increase in DNA per cell remains unclear.

### 3.7. AEs

Four of the included articles investigated the safety of exogenous IL-7, but their data were not available for meta-analysis [26–28,31]. IL-7 was ‘well tolerated,’ and most AEs were grade ≤1 and 2, occasionally reaching grade 3 and no grade 4 [27,28,31]. AEs in grades 1 or 2 were mainly injection-related local erythema (53.8%), grade 1 lymphadenopathy (7.5%), grade 1 fever (2.5%), rash of grade 1 or 2 (2.4%), and fatigue of grade 1 (3.6%) [31]. Grade 3 AEs included hypophosphatemia (9.2%) [31], right lower limb (6.7%) [26], and elevated liver function enzymes (10%) [28].

## 4. Discussion

The advent of ART has significantly improved the lives of individuals with HIV. However, eradicating HIV remains challenging due to the depletion of CD4^+^ T cells in the immune system and the formation of a latent reservoir of the virus integrated into CD4^+^ T cells [34]. Therefore, IL-7 has been proposed as an adjuvant to restore the immune system. In this systematic review and meta-analysis, we observed that IL-7 contributed to an increase in CD4^+^ and CD8^+^ T cell counts in individuals with HIV receiving long-term ART with effective control of the viral load. Additionally, the whole-blood HIV DNA load increased, whereas the HIV DNA load in CD4^+^ T cells and peripheral PBMCs did not change. Additionally, IL-7 was well tolerated [29].

When infected with HIV, the primary target of the virus, or CD4^+^ T cells, are attacked, leading to a rapid decline in cell count and resulting in compromised immunity and a variety of serious life-threatening complications [35,36]. ART significantly inhibits viral replication and reduces morbidity and mortality in individuals with HIV [37]. However, in a subset of individuals undergoing long-term ART, CD4^+^ T cell counts fail to rebound despite decreased viral load, which may be associated with higher mortality rates [38,39]. In addition, the few HIV that integrate into CD4^+^ T cells and PMBCs, known as the ‘latent reservoir,’ can rapidly replicate once ART is discontinued and cannot be eradicated [40]. Therefore, the studies included in this meta-analysis suggest that the administration of IL-7, in combination with ART can boost CD4^+^ T cell counts [26] and activate or enhance HIV DNA load [31].

First, we observed significant increases in CD4^+^ T cell counts at weeks 4 and 12 after IL-7 administration in individuals with HIV who received long-term ART. Katlama et al. [26] found that after IL-7 administration, although CD4^+^ T cell counts peaked at week 12 and gradually decreased, they remained higher than the baseline at week 56, likely due to the half-life period. In another study [31], IL-7 was administered at the beginning of each three-month cycle, and CD4^+^ T cell counts were measured at the end of each cycle. If CD4^+^ T cell counts were < 550/µL, IL-7 administration was continued in the next cycle. The study found that CD4^+^ T cell counts were maintained at >550/µL for all participants by the end of the fourth cycle. These studies showed that the continuous administration of IL-7 could maintain a sufficient CD4^+^ T cell count in individuals with HIV undergoing long-term ART. Additionally, during the expansion of CD4^+^ T cell compartments, central memory and naïve T cell populations were significantly elevated [26–30]. In addition to the circulation, CD4^+^ T cell counts increase in the gut mucosa after IL-7 administration [30]. Notably, IL-2 could increase CD4^+^ T cell counts in HIV-positive adults when combined with ART. However, IL-2 did not provide any significant benefit in terms of mortality, and there was a potential increase in grade 3 or 4 AEs [11].

The increase in CD4^+^ T cells reflects the expansion of the T cell compartment. Therefore, CD8^+^ T cells, another major group of the T cell compartments, and HIV-specific CD8^+^ T cells are of major significance in ART. Thus, the number of CD8^+^ T cells should also be evaluated [41,42]. Our meta-analysis revealed that 20 μg/Kg of IL-7 increased CD8^+^ T cell counts at weeks 4 and 12, suggesting that supplementation with 20 μg/Kg IL-7 can increase CD8^+^ T cell counts in individuals with HIV on long-term ART. Studies have shown that CD8^+^ T cell counts increase on day 14, peak at week 12, and gradually decrease at weeks 56 and 80, but remain above baseline levels [26,27]. Maintaining IL-7 concentrations in the blood may help maintain CD8^+^ T cell counts at a high level; however, sufficient clinical trials are needed to validate this hypothesis. In addition, IL-21, a pleiotropic cytokine, promotes the functional differentiation of multiple CD4^+^ T cell subsets and stimulates the proliferation and functional response of CD8^+^ T cells [43]. Exogenously increasing IL-21 or promoting pre-existing IL-21 cell pools may help stimulate immune resistance to the initial HIV-1 infection [44].

In addition, HIV DNA load is a fundamental indicator of HIV infection [45]. Given that IL-7 can increase CD4^+^ and CD8^+^ T cell counts in individuals with HIV receiving long-term ART, its effect on HIV DNA load is also noteworthy. Our meta-analysis revealed that HIV DNA load in whole blood increased at weeks 4 and 12 after administration of 20 μg/Kg IL-7, although the HIV DNA load in CD4^+^ T cells and PMBCs did not change. Katlama et al. [26] reported that the HIV DNA load in whole blood at weeks 56 and 80 after IL-7 administration was lower than that at week 12, although it was still higher than the baseline. Notably, the HIV DNA load in PBMCs showed a downward trend from baseline at weeks 56 and 80 after IL-7 administration [26]. The increased HIV DNA load in whole blood may be due to the activation of T cells, especially central memory and naïve T cells, as IL-7 promotes the activation of the latent HIV reservoir. This is similar to the results of the Machine learning study by Semenova et al. [46].

IL-7 plays an important role in the development and preservation of memory immune cell function, which provides a strong theoretical basis for the IL-7 administration in the adjuvant treatment of HIV [47]. Despite the beneficial effect of IL-7 in promoting an increase in CD4^+^ T and CD8^+^ T cell counts, another study has shown that IL-7 therapy may increase viral production in areas such as the gut during ART. This could contribute to ongoing inflammation and HIV persistence, leading to a 70% increase in the absolute number of circulating CD4^+^ T cells harboring integrated HIV DNA 4 weeks post-therapy [48]. This may be because IL-7 activates latent HIV virus in T cells while boosting the number of T cells. On the one hand, IL-7 may reconstitute the host immune system by enhancing the immune response of T cells. On the other hand, enhanced IL-7 signaling may help activate latent HIV reservoir, reduce HIV latency in the immune system, and accelerate viral clearance. The rational and effective combination of IL-7 and antiviral drugs can not only maintain the immune function, but also promote the clearance of viral latent reservoir, which may be used as a ‘kick and kill’ strategy to replace traditional ART. Notably, IL-7 induced increases in HIV DNA load may enhance viral replication and risk viral rebound, highlighting the need for long-term monitoring and further clinical studies to assess safety and treatment implications.

This study found that IL-7 can promote CD4^+^ T cell recovery in individuals receiving ART who experience incomplete immune reconstitution. In particular, a weekly dose of 20 µg/kg is recommended, highlighting the potential of IL-7 as an adjunctive therapy. Integrating IL-7 into current ART regimens might improve immune recovery and reduce infection risk. During adjunctive IL-7 therapy with ART, close monitoring of CD4^+^ T cell counts, HIV DNA load, and immune-related AEs is recommended to guide individualized treatment. While IL-7 shows promise, potential risks, such as transient increases in HIV DNA load or immune activation, should be considered. Although IL-7 requires intramuscular injection, adverse effects are rare and mild, suggesting that compliance should be high [49]. However, the production and storage of IL-7 require complex biotechnology facilities, which leads to elevated treatment costs. Moreover, as an immunomodulator, IL-7 requires well-equipped medical facilities and professional personnel to administration [50]. Therefore, although IL-7 is easy to administer, its high cost and the need for well-equipped healthcare facilities may pose challenges for implementation in resource-limited regions, suggesting that initial deployment could focus on areas with more advanced medical infrastructure. These findings provide evidence to optimize IL-7 administration in ART-treated individuals. Further large-scale, randomized studies are warranted to confirm long-term efficacy and safety, refine dosing protocols, and determine the optimal timing of IL-7 integration into ART.

## 5. Conclusions

This systematic review and meta-analysis is the first to show that IL-7 can increase CD4^+^ and CD8^+^ T cell counts, as well as HIV DNA load, in HIV individuals who are virus-suppressed on long-term ART. However, due to the complex role of IL-7 in immune reconstitution, careful evaluation is needed before clinical application. Additional long-term studies are needed to explore how factors such as baseline T cell counts and ART duration affect the effects of IL-7. These findings may inform ART guidelines by emphasizing the potential of IL-7 to enhance immune function, but its integration into standard treatment regimens should be considered on a case-by-case basis rather than for routine use.

## Supplementary Material

Supplemental Material

## Data Availability

The original contributions presented in the study are included in the article and Supplementary Material; further inquiries can be directed to the corresponding author.
