# Supplementary material for: Adjuvant roles of interleukin-7 in enhancing T cell recovery during antiretroviral therapy for individuals with HIV: a systematic review and meta-analysis
Source: Ann Med. 2025 Dec 1;57(1):2594303. doi: 10.1080/07853890.2025.2594303 (PMC12671416; doi:10.1080/07853890.2025.2594303)
Supplement: Supplemental Material [file IANN_A_2594303_SM4541.zip › suppl_data/Supplementary material Figure caption.docx]

**Additional Files**

**Supplementary Figure 1.** Risk of bias in included studies. Green represents low risk, yellow represents unclear risk, and red represents high risk of bias.

**Supplementary Figure 2.** Meta-analysis of CD4^+^ T cell counts at week 4 after IL-7 administration and before administration in individuals with HIV receiving long-term ART. ART, antiretroviral therapy; Chi^2^, Chi-square test; CI, confidence interval; df, degrees of freedom; IL-7, Interleukin-7; IV, inverse variance; I^2^, I-squared; SD, standard deviation; Std., standard deviation; W 4, week 4.

**Supplementary Figure 3.** Meta-analysis of CD8^+^ T cell counts at week 4 after IL-7 administration and before administration in individuals with HIV receiving long-term ART. ART, antiretroviral therapy; Chi^2^, Chi-square test; CI, confidence interval; df,

degrees of freedom; IL-7, Interleukin-7; IV, inverse variance; I^2^, I-squared; SD, standard deviation; Std., standard deviation; W 4, week 4.

**Supplementary Figure 4.** Meta-analysis of HIV DNA load in whole blood at week 4 after IL-7 administration and before administration in individuals with HIV receiving long-term ART**.** ART, antiretroviral therapy; Chi^2^, Chi-square test; CI, confidence interval; df, degrees of freedom; IL-7, Interleukin-7; IV, inverse variance; I^2^, I-squared; SD, standard deviation; W 4, week 4.

**Supplementary Figure 5.** Meta-analysis of HIV DNA load in CD4^+^ T cells at week 12 after IL-7 administration and before administration in individuals with HIV receiving long-term ART. ART, antiretroviral therapy; Chi^2^, Chi-square test; CI, confidence interval; df, degrees of freedom; IL-7, Interleukin-7; IV, inverse variance; I^2^, I-squared; SD, standard deviation; W 12, week 12.

**Supplementary Figure 6.** Meta-analysis of HIV DNA load in PMBCs at week 12 after IL-7 administration and before administration in individuals with HIV receiving long-term ART. ART, antiretroviral therapy; Chi^2^, Chi-square test; CI, confidence interval; df, degrees of freedom; IL-7, Interleukin-7; IV, inverse variance; I^2^, I-squared; SD, standard deviation; W 12, week 12.

**Supplementary Figure 7.** Meta-analysis of HIV DNA load in CD4^+^ T cells at week 4 after IL-7 administration and before administration in individuals with HIV receiving long-term ART. ART, antiretroviral therapy; Chi^2^, Chi-square test; CI, confidence interval; df, degrees of freedom; IL-7, Interleukin-7; IV, inverse variance; I^2^, I-squared; SD, standard deviation; W 4, week 4.

**Supplementary Figure 8.** Meta-analysis of HIV DNA load in PMBCs at week 4 after IL-7 administration and before administration in individuals with HIV receiving long-term ART. ART, antiretroviral therapy; Chi^2^, Chi-square test; CI, confidence interval; df, degrees of freedom; IL-7, Interleukin-7; IV, inverse variance; I^2^, I-squared; SD, standard deviation; W 4, week 4.
