# Supplementary figures and images for: Adjuvant roles of interleukin-7 in enhancing T cell recovery during antiretroviral therapy for individuals with HIV: a systematic review and meta-analysis
Source: Ann Med. 2025 Dec 1;57(1):2594303. doi: 10.1080/07853890.2025.2594303 (PMC12671416; doi:10.1080/07853890.2025.2594303)

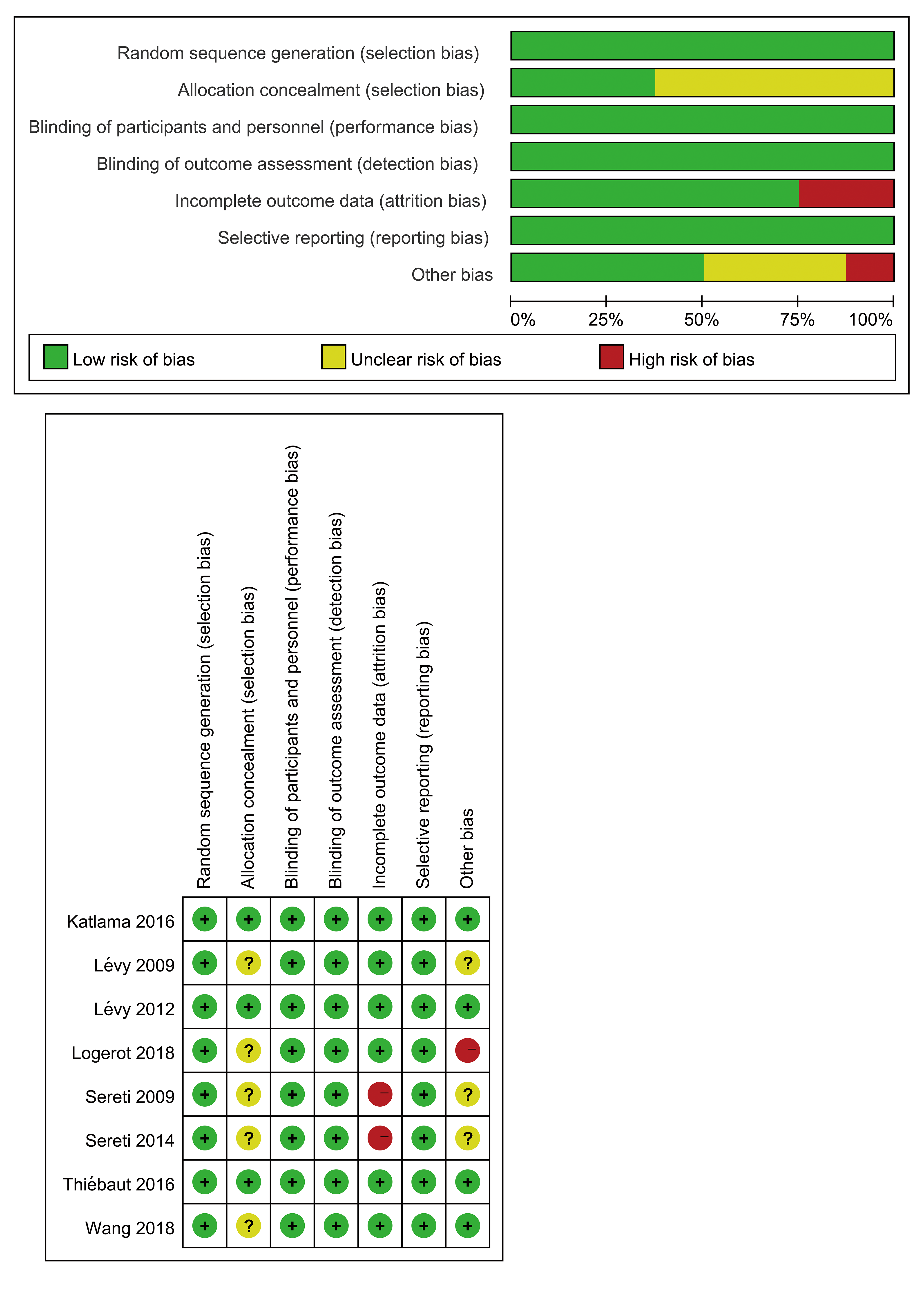

Supplement: Supplemental Material [file IANN_A_2594303_SM4541.zip › suppl_data/Supplementary Figure 1.tif]

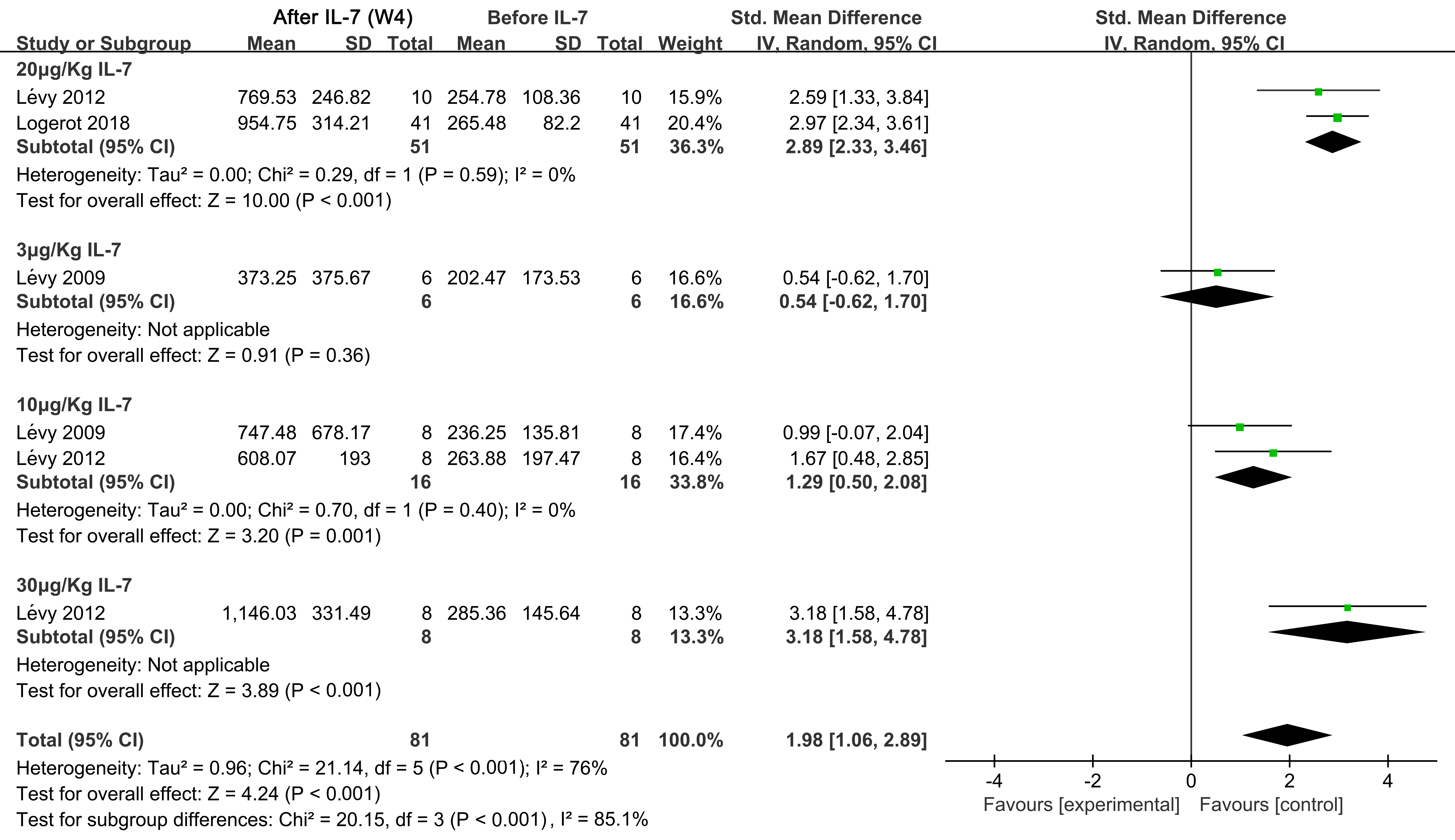

Supplement: Supplemental Material [file IANN_A_2594303_SM4541.zip › suppl_data/Supplementary Figure 2.tif]

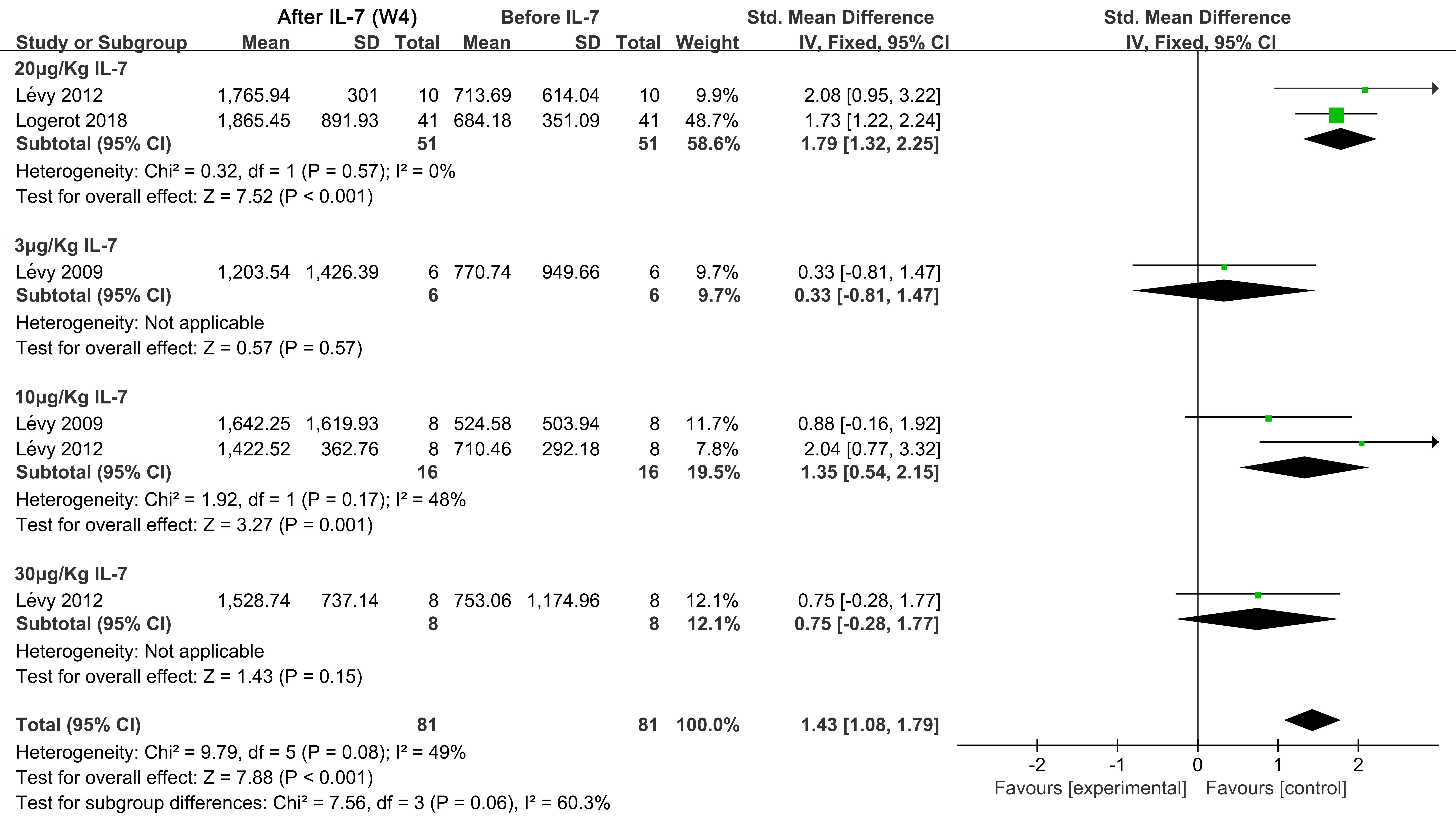

Supplement: Supplemental Material [file IANN_A_2594303_SM4541.zip › suppl_data/Supplementary Figure 3.tif]

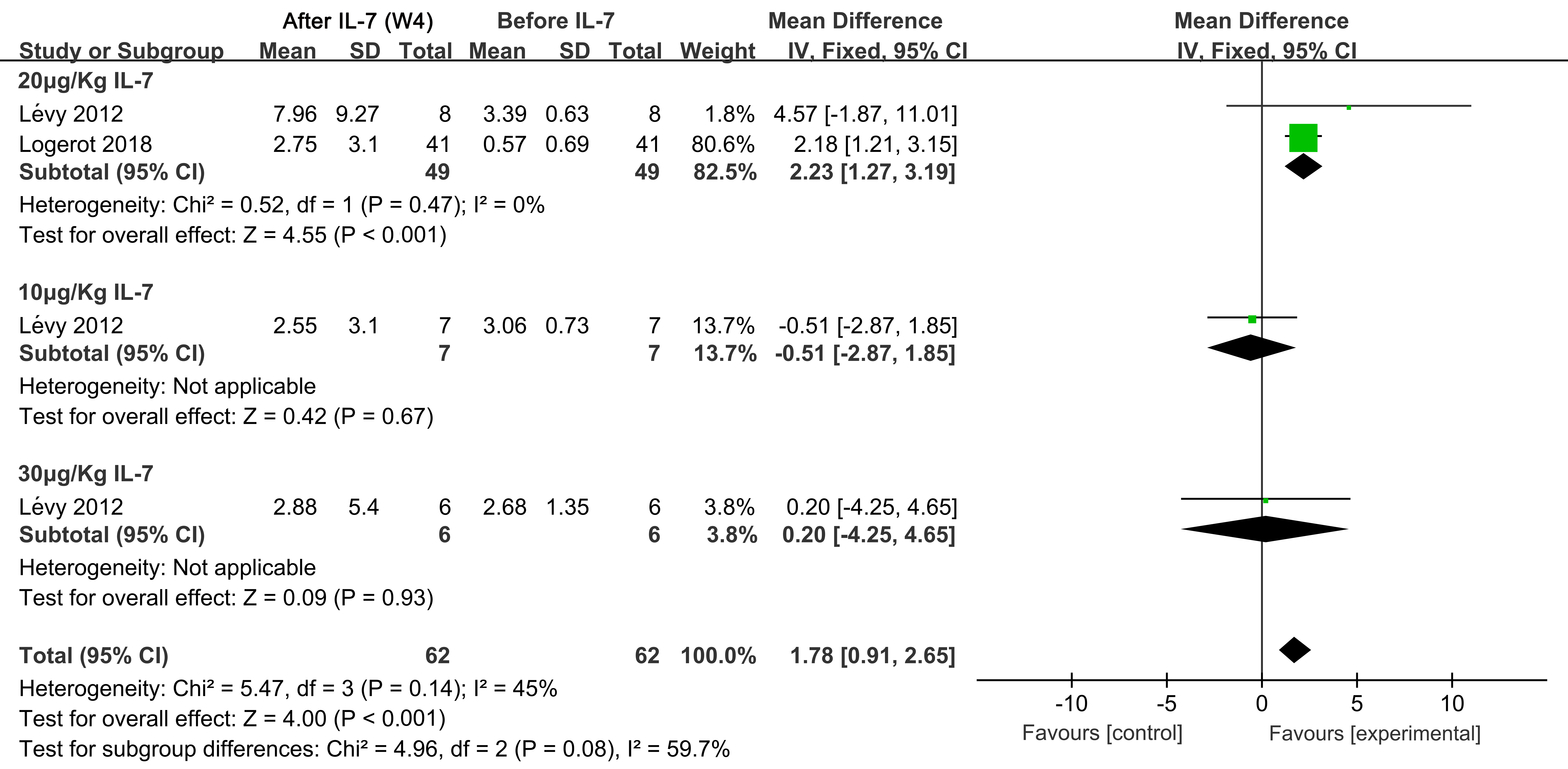

Supplement: Supplemental Material [file IANN_A_2594303_SM4541.zip › suppl_data/Supplementary Figure 4.tif]

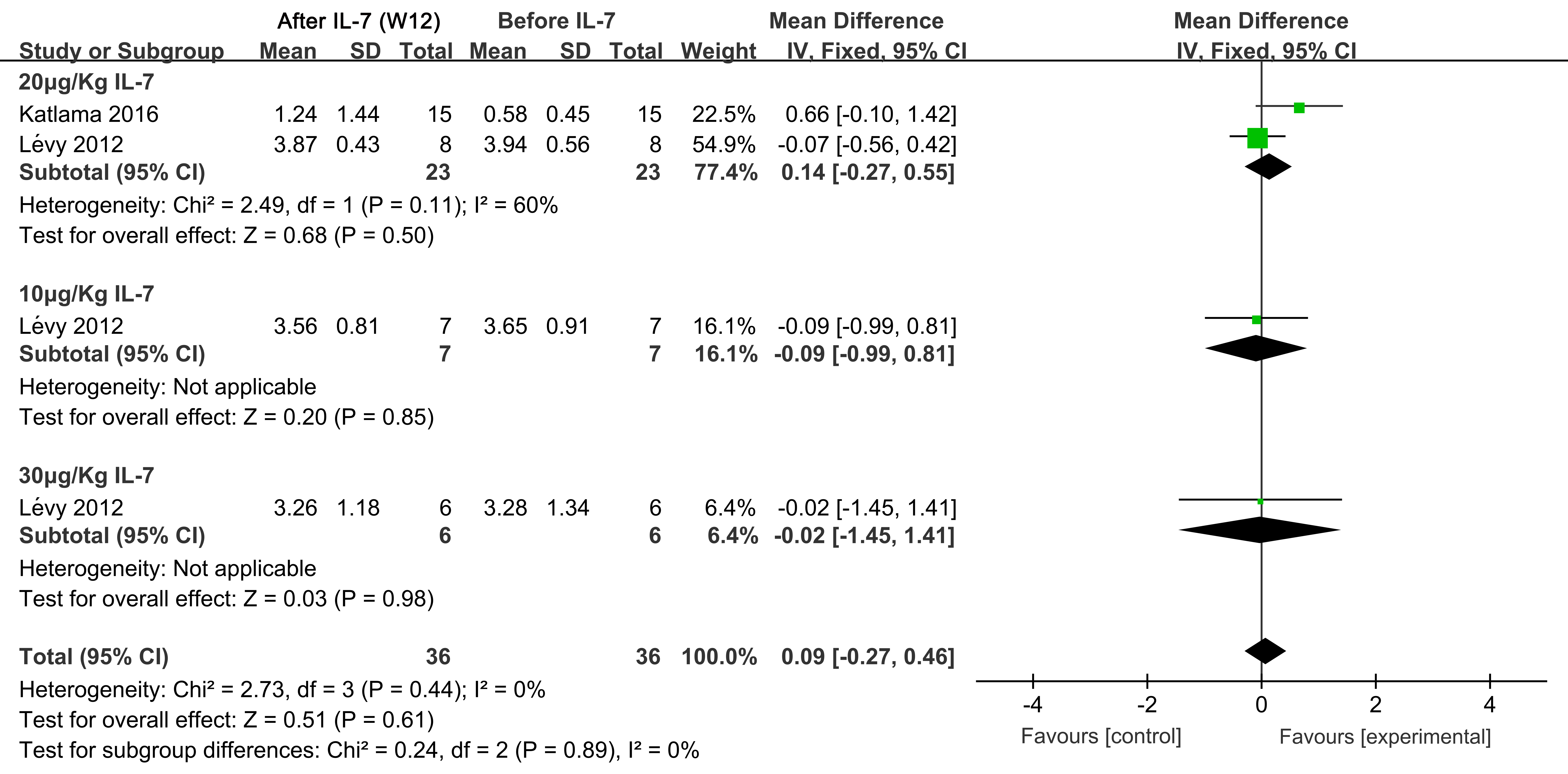

Supplement: Supplemental Material [file IANN_A_2594303_SM4541.zip › suppl_data/Supplementary Figure 5.tif]

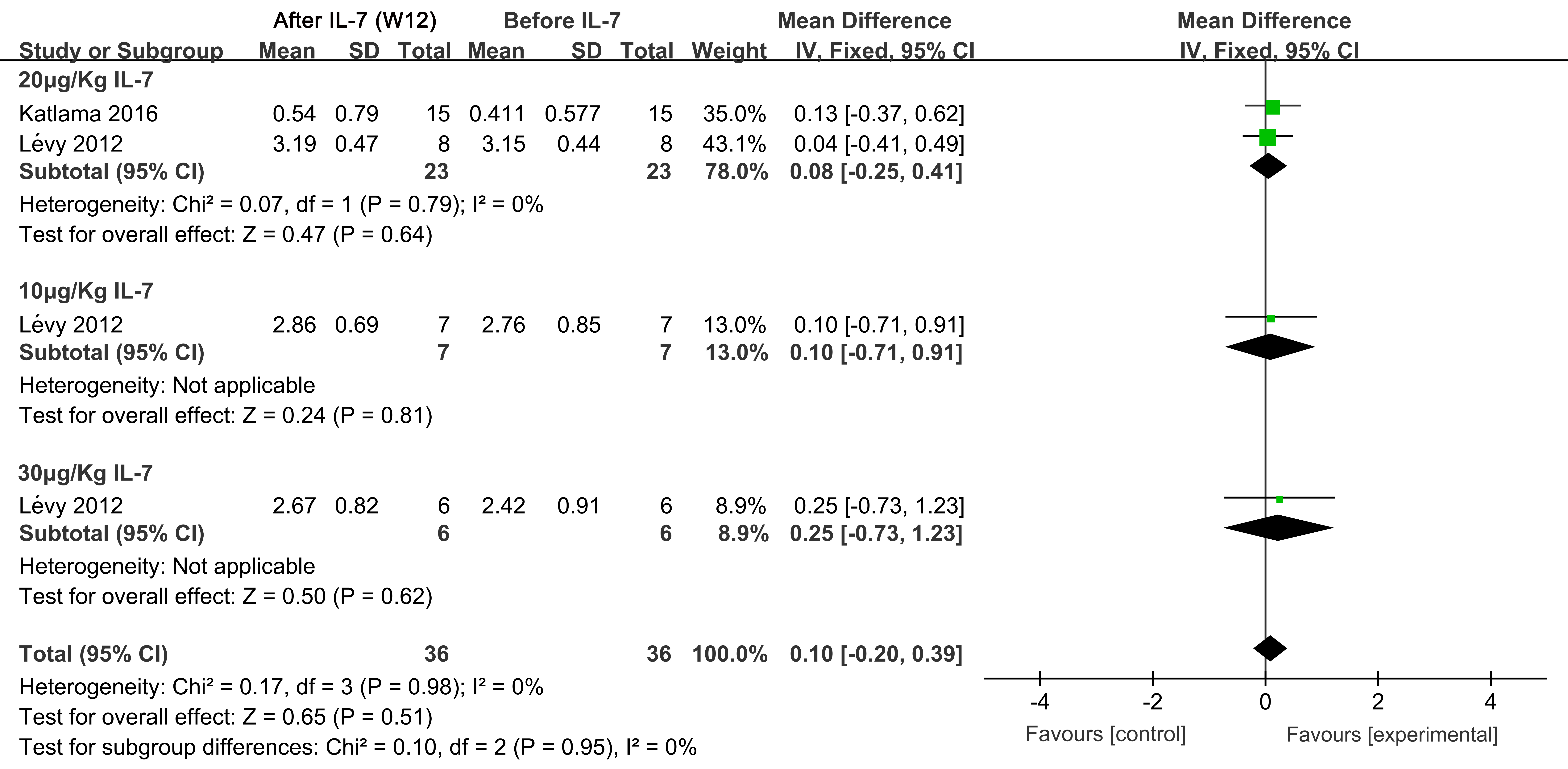

Supplement: Supplemental Material [file IANN_A_2594303_SM4541.zip › suppl_data/Supplementary Figure 6.tif]

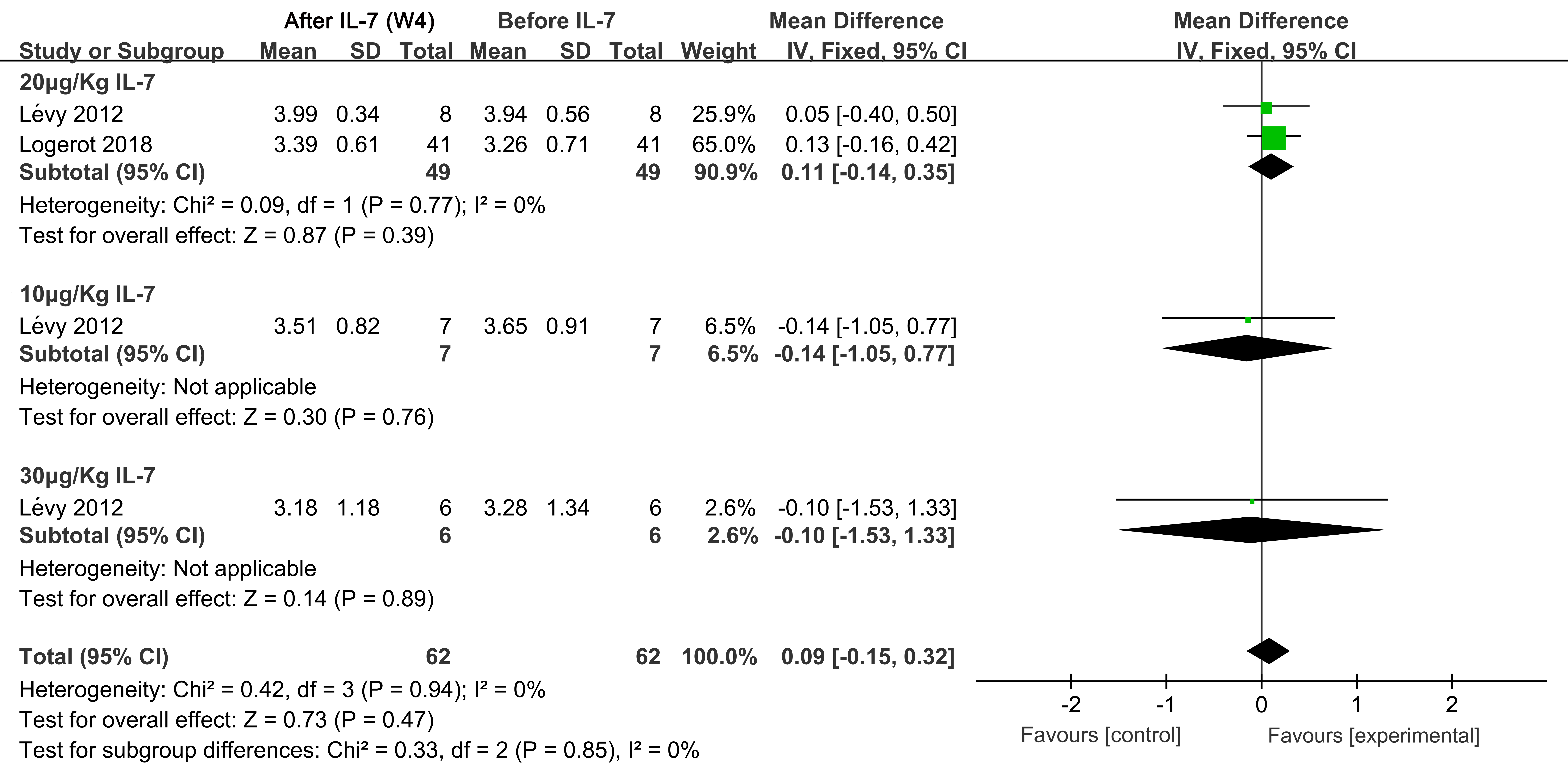

Supplement: Supplemental Material [file IANN_A_2594303_SM4541.zip › suppl_data/Supplementary Figure 7.tif]

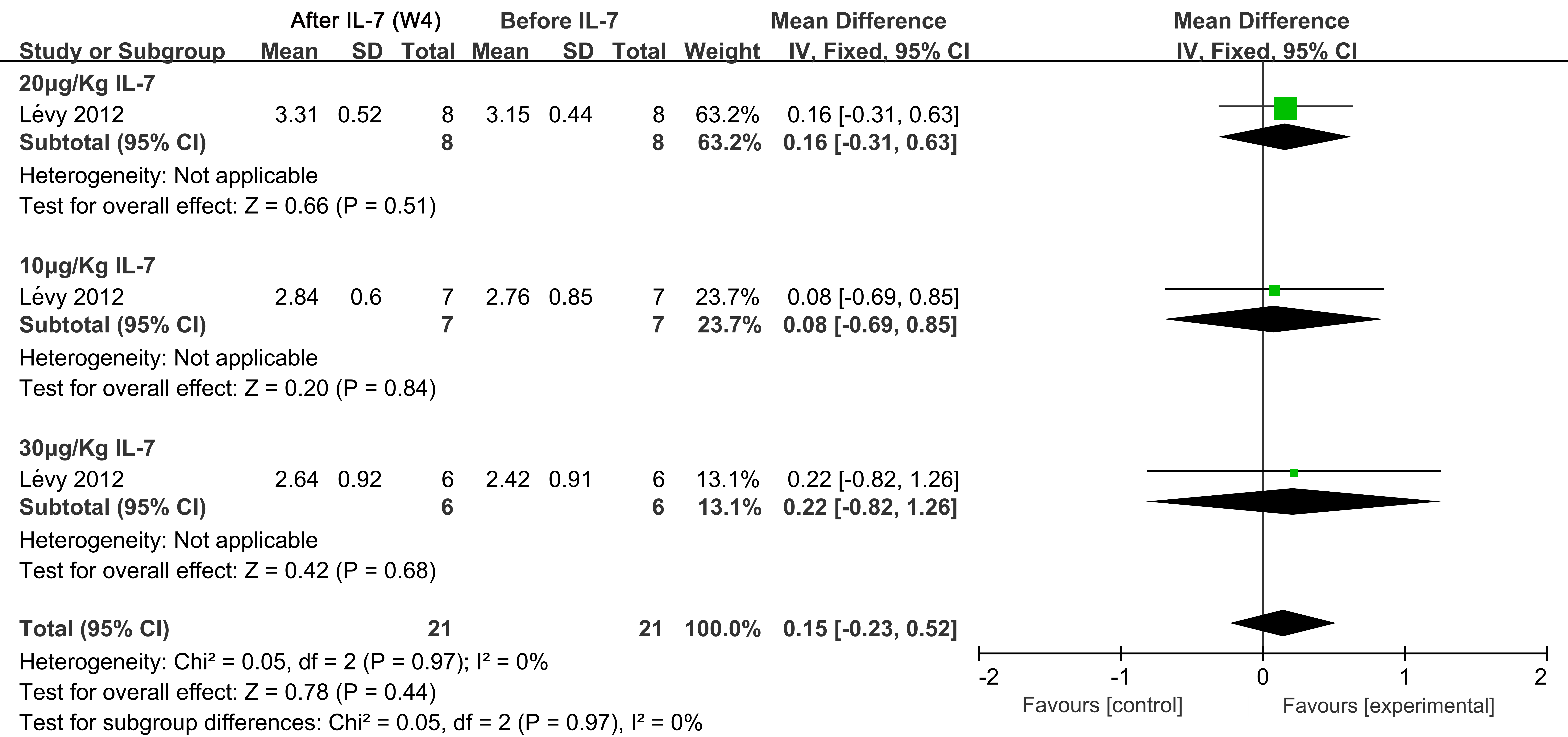

Supplement: Supplemental Material [file IANN_A_2594303_SM4541.zip › suppl_data/Supplementary Figure 8.tif]
