# Supplementary material for: Adjuvant roles of interleukin-7 in enhancing T cell recovery during antiretroviral therapy for individuals with HIV: a systematic review and meta-analysis
Source: Ann Med. 2025 Dec 1;57(1):2594303. doi: 10.1080/07853890.2025.2594303 (PMC12671416; doi:10.1080/07853890.2025.2594303)
Supplement: Supplemental Material [file IANN_A_2594303_SM4541.zip › suppl_data/Supplementary material Complete search strategy.doc]

**Complete search strategy**

("interleukin 7"[MeSH Terms] OR "interleukin 7"[All Fields] OR "il 7"[All Fields] OR ("interleukin 7"[MeSH Terms] OR "interleukin 7"[All Fields] OR "interleukin 7"[All Fields])) AND ("hiv"[MeSH Terms] OR "hiv"[All Fields] OR ("hiv"[MeSH Terms] OR "hiv"[All Fields] OR ("human"[All Fields] AND "immunodeficiency"[All Fields] AND "virus"[All Fields]) OR "human immunodeficiency virus"[All Fields]))
